# Microglia use multiple mechanisms to mediate interactions with vitronectin; non-essential roles for the highly-expressed αvβ3 and αvβ5 integrins

**DOI:** 10.1186/1742-2094-8-157

**Published:** 2011-11-10

**Authors:** Jennifer V Welser-Alves, Amin Boroujerdi, Ulrich Tigges, Richard Milner

**Affiliations:** 1 Department of Molecular and Experimental Medicine, The Scripps Research Institute, 10550 North Torrey Pines Road, La Jolla, CA 92037, USA

**Keywords:** microglia, extracellular matrix, vitronectin, integrin, adhesion, MMP-9

## Abstract

**Background:**

As the primary resident immune cells, microglia play a central role in regulating inflammatory processes in the CNS. The extracellular matrix (ECM) protein vitronectin promotes microglial activation, switching microglia into an activated phenotype. We have shown previously that microglia express two vitronectin receptors, αvβ3 and αvβ5 integrins. As these integrins have well-defined roles in activation and phagocytic processes in other cell types, the purpose of the current study was to investigate the contribution of these two integrins in microglial activation.

**Methods:**

Microglial cells were prepared from wild-type, β3 integrin knockout (KO), β5 integrin KO or β3/β5 integrin DKO mice, and their interactions and activation responses to vitronectin examined in a battery of assays, including adhesion, expression of activation markers, MMP-9 expression, and phagocytosis. Expression of other αv integrins was examined by flow cytometry and immunoprecipitation.

**Results:**

Surprisingly, when cultured on vitronectin, microglia from the different knockout strains showed no obvious defects in adhesion, activation marker expression, MMP-9 induction, or phagocytosis of vitronectin-coated beads. To investigate the reason for this lack of effect, we examined the expression of other αv integrins. Flow cytometry showed that β3/β5 integrin DKO microglia expressed residual αv integrin at the cell surface, and immunoprecipitation confirmed this finding by revealing the presence of low levels of the αvβ1 and αvβ8 integrins. β1 integrin blockade had no impact on adhesion of β3/β5 integrin DKO microglia to vitronectin, suggesting that in addition to αvβ1, αvβ3, and αvβ5, αvβ8 also serves as a functional vitronectin receptor on microglia.

**Conclusions:**

Taken together, this demonstrates that the αvβ3 and αvβ5 integrins are not essential for mediating microglial activation responses to vitronectin, but that microglia use multiple redundant receptors to mediate interactions with this ECM protein.

## Background

Microglia are immune effector cells resident in the central nervous system (CNS), whose main role is to orchestrate immunological responses following cerebral insults [1-3]. In the resting CNS, microglia occupy a basal surveillance state, but after activation by pro-inflammatory cytokines or microorganisms, they transform into metabolically active phagocytic cells, upregulating expression of cytokines and chemokines, and migrating to the inflammatory focus. As well as playing a protective role, recent evidence suggests that in some diseases, including multiple sclerosis (MS), microglia may become inappropriately stimulated, leading to auto-immune destruction of host tissue [4-7].

To understand why microglia may become inappropriately activated in the early stages of MS, we have focused our attention on the function of certain ECM proteins present in blood at high concentrations, including fibronectin and vitronectin [8,9]. We have demonstrated that the plasma proteins vitronectin and fibronectin promote microglial activation in vitro [10,11]. Taken together with the observation that blood-brain barrier (BBB) breakdown is an early event in the pathogenesis of MS we proposed that leakage of these two proteins into brain parenchymal tissue pre-disposes to microglial activation and myelin damage. Results obtained with the experimental autoimmune encephalomyelitis (EAE) model demonstrated that BBB breakdown was closely associated with fibronectin and vitronectin deposits in the CNS, which closely correlated with microglial activation and expression of the matrix metalloproteinase, MMP-9 [11]. Combined with the evidence from other groups demonstrating vitronectin and fibronectin deposition in demyelinated lesions in the brains of MS patients [12-14] and EAE mice [15], this supports the hypothesis that fibronectin and vitronectin promote microglial activation in vivo.

A major question yet to be fully answered is: which microglial receptors mediate the activation response to vitronectin? Our prior work has shown that microglia express the two vitronectin receptors, αvβ3 and αvβ5 integrins, and that the microglial response to vitronectin is largely mediated by αv integrins [11]. As the αvβ3 and αvβ5 integrins have well-defined roles in activation and phagocytic processes in other cell types, the purpose of the current study was to investigate the contribution of these two integrins in this process, and thereby test our hypothesis that absence of both αvβ3 and αvβ5 integrins would render microglia unresponsive to vitronectin. To examine these events, microglial cells were prepared from wild-type, β3 integrin KO, β5 integrin KO and β3/β5 integrin DKO mice, and the behavior of these microglia evaluated in a battery of assays including cell adhesion, expression of activation markers and MMP-9, and phagocytosis of vitronectin-coated beads.

## Methods

### Animals

The studies described have been reviewed and approved by The Scripps Research Institute Institutional Animal Care and Use Committee. β3 integrin KO and β5 integrin KO mice (backcrossed > 10 times on the C57Bl/6 background) were maintained under pathogen-free conditions in the closed breeding colony of The Scripps Research Institute (TSRI). β3 integrin KO and β5 integrin KO mice were bred and offspring genotyped using previously described protocols [16-19] to generate homozygous β3 integrin KO (β3 -/-), homozygous β5 integrin KO (β5 -/-), and double-knockout (DKO) mice, homozygous (β3 -/-, β5 -/-). In all experiments, littermate wild-type mice were used as controls.

### Cell culture

Pure cultures of mouse microglia were obtained as described previously [20], with cultures from the different strains of mice being established at the same time in parallel. Briefly, 7-10 day old mixed glial cultures were shaken for 30 minutes and the supernatant containing detached microglia was collected. Microglia were counted by hemocytometer and plated at a density of 2 × 10^5 ^cells/well in six-well plates (Nunc, Naperville, IL) previously coated for two hours at 37°C with a 10 μg/ml solution of vitronectin (Sigma). Cells were grown overnight in the mixed glial culture media, and then switched to N1 serum-fee media (DMEM supplemented with N1 (Sigma). The purity of these microglial cultures was > 99% as determined by Mac-1 positivity in flow cytometry.

### Cell adhesion assays

Adhesion assays were performed as previously described [20]. Briefly, substrates were prepared by coating the central area of 24 well plates (Nunc) with 25 μl of ECM solution (10 μg/ml of vitronectin or fibronectin, both from Sigma) for 2 hours at 37°C. Substrates were washed twice before addition of cells. Microglia were prepared as described above, centrifuged, re-suspended in N1 serum-free media, and 2000 microglia applied to the substrates in a 25 μl drop and then incubated at 37°C for 15 or 30 minutes. In the function-blocking experiments, the anti-αv monoclonal antibody (RMV-7), anti-β1 monoclonal antibody (Ha2/5) or control antibodies were included at a concentration of 5 μg/ml. The assay was stopped by adding 1 ml of DMEM and washing off any loosely attached cells. The attached cells were fixed in 4% paraformaldehyde in PBS for 20 minutes, and stored in PBS. Adhesion was quantified under phase microscopy by counting all attached cells within 5 fields of view per condition. Within each experiment each condition/time-point was performed in duplicate; the results represent the mean ± SEM of three experiments. Statistical significance was assessed by using the Student's paired t test, in which p < 0.05 was defined as statistically significant.

### Antibodies

The following monoclonal antibodies were obtained from BD Pharmingen (La Jolla, CA): rat monoclonal antibodies reactive for MHC class I (M1/42.3.9.8), the integrin subunits α4 (MFR4.B), α5 (5H10-27 (MFR5)), αv (RMV-7), αM (M1/70), and the isotype control antibody, rat anti-KLH (A110-2), and the hamster monoclonal antibodies reactive for the β1(Ha2/5; function-blocking antibody) and β3 (2C9.G2) integrin subunits and isotype control (G235-1). Rabbit polyclonal antibodies specific for the αv integrin subunit were obtained from Chemicon (Temecula, CA). The anti-β8 integrin polyclonal antibody was a kind gift from Dr. Joseph McCarty, M.D. Anderson Cancer Center, Houston, TX.

### Cell surface labeling and immunoprecipitation

Microglial cell surface molecules were labeled with biotin as previously described [10,21]. Briefly, microglial cell cultures were incubated with NHS-LC-biotin (Pierce, Rockford, IL) for 30 minutes, washed in TRIS-containing cell wash buffer (CWB), and then removed from tissue culture plates and centrifuged. Cells were lysed in 0.5% Triton-X100 in CWB that contained a cocktail of protease inhibitors (Invitrogen, Carlsbad, CA). After 30 minutes on ice, the lysate was centrifuged to remove the insoluble fraction. The supernatants were pre-cleared for one hour with 30 μl of protein A sepharose or protein G sepharose per ml of cell lysate. Immunoprecipitations were performed overnight at 4°C on a rotating platform using the polyclonal anti-αv or anti-β8 integrin antibodies at 1:250 dilution in a tube containing 30 μl protein A sepharose. Beads were washed 5 times in immunoprecipitation wash buffer, as previously described and the integrin immune complexes were separated by boiling the beads in non-reducing sample buffer for 5 minutes before being analysed by 8% SDS-PAGE (Invitrogen) under non-reducing conditions. Proteins were electro-blotted for 1.5 hours onto nitrocellulose membranes (Invitrogen), blocked overnight in 3% BSA in TBS containing 0.1% Tween-20 (Sigma) and probed with streptavidin-HRP conjugate (Pierce) for one hour, before being extensively washed. Protein bands were visualised with the SuperSignal WestFemto ECL detection system (Pierce) according to the manufacturers' instructions.

### Microglial phagocytosis of vitronectin-coated beads

Microglial cells were plated at a density of 2 × 10^5 ^cells/well in six-well plates. After one day of culture, 2.5 μl of a suspension of yellow-green fluorescent beads (Molecular Probes, Eugene, OR) previously coated in a 100 μg/ml vitronectin solution for 2 hours at 37°C, was added to the microglia, and thoroughly mixed with the tissue culture media to distribute the beads throughout the culture. After a further 24 hours, cultures were visualised for microglial uptake of beads, and microglia collected and phagocytic uptake of fluorescent beads analyzed by flow cyometry, with 10,000 events recorded for each condition. The phagocytic index of microglia was quantified and expressed as the mean fluorescent intensity of the cell population. Each experiment was repeated a minimum number of four times and the data expressed as mean ± SD. Statistical significance was assessed by using the Student's paired t test, in which p < 0.05 was defined as statistically significant.

### Flow cytometry

Microglia, isolated from the four different strains of mice, were cultured in vitronectin-coated 6-well plates under serum-free conditions. After 2 days, microglia were removed from the culture plates and cell surface expression of MHC class I and the integrins α4, α5, αv or Mac-1 analyzed by flow cytometry using direct fluorescent-conjugated monoclonal antibodies, as described previously [20]. The fluorescent intensity of the labeled cells was analyzed with a Becton Dickinson FACScan machine (San Diego, CA), with 10,000 events recorded for each condition. For each experimental condition, the mean fluorescent intensity was compared with the control state and expressed as the percentage change relative to the control condition. Each experiment was repeated a minimum number of four times and the data expressed as mean ± SD. Statistical significance was assessed by using the Student's paired t test, in which p < 0.05 was defined as statistically significant.

### Gel zymography

Gelatin zymography was used to detect MMP-9 activity as previously described [11,22]. Microglial cells were plated at a density of 2 × 10^5 ^cells/well in six-well plates that were either left uncoated, or coated with vitronectin or fibronectin. After 2 days culture, microglial supernatants were collected and analyzed for gelatinolytic activity. Positive controls for MMP-9 and MMP-2 (obtained from R&D) were included. For quantification, gels were scanned using a Bio-Rad VersaDoc imaging system (Hercules, CA) and band intensities quantified using the NIH Image program. Each experiment was repeated a minimum number of four times and the data expressed as mean ± SD. Statistical significance was assessed by using the Student's paired t test, in which p < 0.05 was defined as statistically significant.

## Results

### Absence of αvβ3, αvβ5, or both integrins does not diminish microglial adhesion to vitronectin

**Figure 1:**
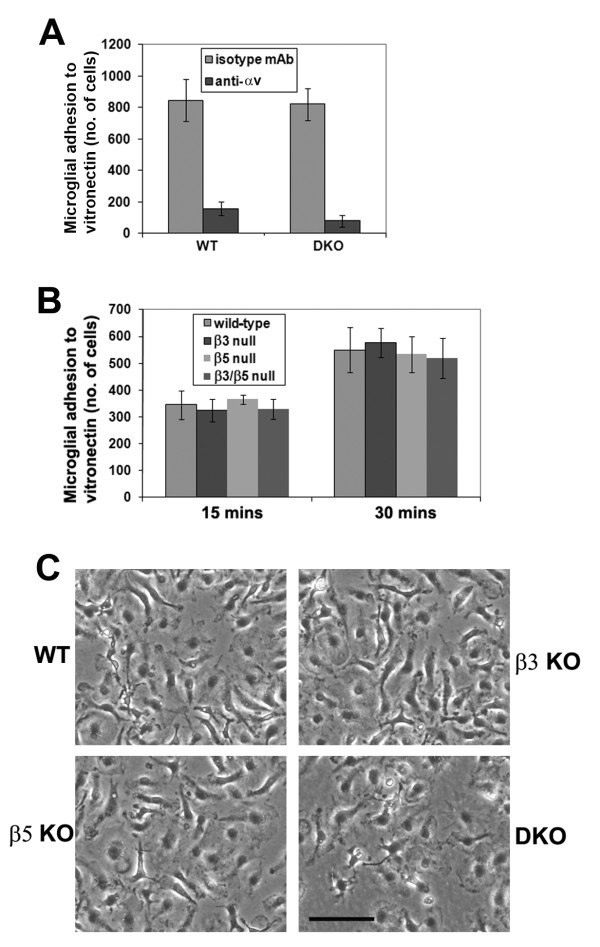
**Evaluating the role of αv integrins in mediating microglial adhesion to vitronectin**. A. Adhesion to vitronectin of microglia derived from wild-type or β3/β5 integrin DKO mice in the presence or absence of an anti-αv blocking antibody was examined as described in Materials and Methods. Adhesion is expressed as the number of cells adherent within a given field of view after 30 minutes adhesion. All points represent the mean ± SEM of three experiments. B. Time course of adhesion to vitronectin for microglia derived from wild-type, β3 integrin KO, β5 integrin KO and β3/β5 integrin DKO mice. Adhesion is expressed as the number of cells adherent within a given field of view, after 15 and 30 minutes of cell adhesion. All points represent the mean ± SEM of three experiments. C. Phase pictures of wild-type, β3 integrin KO, β5 integrin KO and β3/β5 integrin DKO mice microglia adherent to vitronectin after 60 minutes. Scale bar = 50 μm. Note that none of the KO strains showed defects in their adhesion to vitronectin.

Vitronectin is a strong inducer of microglial activation, and antibody-blocking studies have demonstrated that this effect is mediated primarily via αv integrins [11]. Microglia express high levels of the two vitronectin receptors, αvβ3 and αvβ5 integrins [23], which have well-defined roles in activation and phagocytic processes in other cell types [24,25]. The purpose of the current study was to investigate the contributions of the αvβ3 and αvβ5 integrins to this process, and test our hypothesis that absence of both these integrins would render microglia unresponsive to vitronectin. To examine these events, mixed glial cultures (MGC) were established from postnatal brains of four different strains of mice: wild-type, β3 integrin KO, β5 integrin KO and β3/β5 integrin DKO. In the first set of experiments, we examined the role of αv integrins in mediating microglial adhesion to vitronectin. In 30-minute adhesion assays, an αv integrin function-blocking antibody significantly inhibited the adhesion of both wild-type microglia (from 847 ± 134 cells under control conditions to 157 ± 44 cells with αv antibody, p < 0.005) and β3/β5 integrin DKO microglia (from 822 ± 101 cells under control conditions to 78 ± 39 cells with αv antibody, p < 0.005) to vitronectin (Figure 1A). This demonstrates that αv integrins are the major class of vitronectin receptors that mediate microglial adhesion to vitronectin, confirming the findings from previous studies [11,20]. Next, we examined whether microglia lacking the αvβ3 or αvβ5 integrins can attach to vitronectin. In short term adhesion assays lasting 15 or 30 minutes, we detected no defects in the attachment of any of the knockout strains of microglia compared to wild-type cells (Figure 1B). In addition, after 60 minutes of adhesion, there were no obvious differences in the spreading characteristics or morphology of the different strains (Figure 1C).

### Microglial activation responses to vitronectin are not affected by the absence of αvβ3, αvβ5 or both these integrins

**Figure 2:**
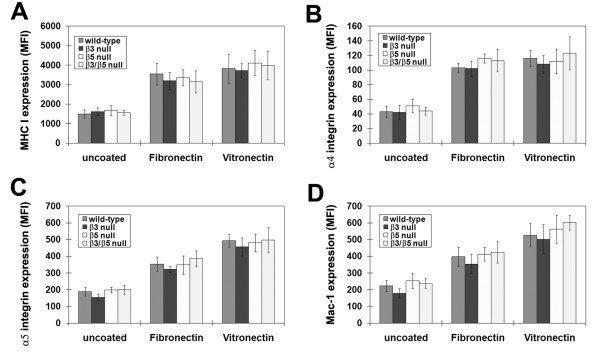
**Evaluating the role of the αvβ3 and αvβ5 integrins in promoting microglial activation state in response to vitronectin**. Wild-type, β3 integrin KO, β5 integrin KO and β3/β5 integrin DKO microglia were purified from mixed glial cultures as described in Materials and Methods, and then cultured in serum-free medium on vitronectin. After two days in culture, microglial expression of the activation marker MHC class I (panel A) or the α4, α5 and αM (Mac-1) integrin subunits (panel B) was analyzed by flow cytometry. All points in the graphs are expressed as the mean fluorescent index (MFI), and represent the mean ± SEM of three experiments. Note that all four strains of microglia expressed equivalent levels of the activation markers, implying that β3 integrin KO, β5 integrin KO and β3/β5 integrin DKO microglia had no defect in their activation response to vitronectin.

Microglial activation correlates with a morphological switch from a phase-bright, process bearing cell to a phase-dark amoeboid phenotype. In light of our finding that β3 KO, β5 KO and β3/β5 DKO show the same morphological activation on vitronectin as wild-type cells (Figure 1B), this suggests that the αvβ3 and αvβ5 integrins are not essential for mediating microglial responses to vitronectin. However, to confirm changes of microglial activation at the molecular level, we also examined cell surface expression of the activation markers MHC class I, and the integrins, α4β1, α5β1 and αMβ2 (Mac-1). Microglia were cultured on vitronectin under serum-free conditions for 2 days, and their expression levels of activation markers quantified by flow cytometry (Figure 2). Consistent with previous results [10], fibronectin and vitronectin stongly promoted microglial expression of all the cell surface markers of activation, including MHC class I and the different activation integrins. However, relative to wild-type cells, microglia lacking β3, β5, or both integrins showed no significant difference in their expression of the activation markers MHC class I, or the integrins, α4β1, α5β1, or Mac-1.

**Figure 3:**
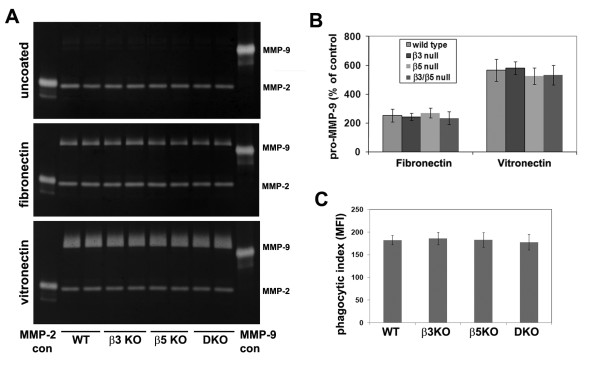
**Examination of the role of the αvβ3 and αvβ5 integrins in mediating vitronectin induction of microglial activation**. Wild-type, β3 integrin KO, β5 integrin KO and β3/β5 integrin DKO microglia were purified from mixed glial cultures as described in Materials and Methods, and then cultured in serum-free medium on uncoated plastic, fibronectin or vitronectin. After 2 days culture, levels of MMP-9 in the microglial supernatants were examined by gel zymography. A. Representative gel zymogram. B. Summary of zymography experiments. Each point is expressed as the percentage change in MMP-9 relative to control (wild-type microglia on uncoated plastic) and represents the mean ± SD of three separate experiments. Note that culture on fibronectin and vitronectin increased MMP-9 expression in microglia from all strains of mice, with no obvious differences detected between wild-type and integrin KO strains on any substrate. C. Examination of the role of the αvβ3 and αvβ5 integrins in mediating microglial phagocytosis. Microglia from all 4 strains were purified as described in Materials and Methods, and cultured in serum-free medium on uncoated plastic for 24 hours before 2 μl of vitronectin-coated yellow-green fluorescent 2 μm beads were added to the cultures. 24 hours later cultures were washed to remove undigested beads and the microglial uptake of fluorescent beads examined by flow cytometry. Each point is expressed as the mean fluorescent index of the microglial population, and represents the mean ± SD of three experiments. Note that none of the integrin null microglia showed defects in their ability to phagocytose vitronectin-coated beads.

As vitronectin strongly promotes microglial expression of the matrix metalloproteinase MMP-9 [11], we also tested whether β3 KO, β5 KO or β3/β5 DKO microglia were deficient in their expression of MMP-9 in response to vitronectin. To quantify microglial expression of MMP-9, gelatin zymography was performed on supernatants taken from microglia cultured under serum-free conditions for three days on vitronectin. Consistent with previous results, fibronectin and vitronectin promoted strong induction of pro-MMP-9 compared to the uncoated plastic control substrate (Figures 3A and 3B) [11]. However, there were no significant differences in the level of MMP-9 induction of any of the KO strains of microglia cultured on vitronectin (or fibronectin), compared with wild-type cells. Next, we evaluated whether there were any defects in the ability of integrin-deficient microglia to phagocytose vitronectin-coated beads. Microglia were incubated with vitronectin-coated fluorescent beads for 24 hours, and the phagocytic uptake of beads analyzed by flow cyometry. As shown in Figure 3C, there were no differences in the phagocytic activity of microglia derived from the different strains of mice.

### Microglia deficient in β3 and β5 integrins show low expression levels of two additional αv integrins

**Figure 4:**
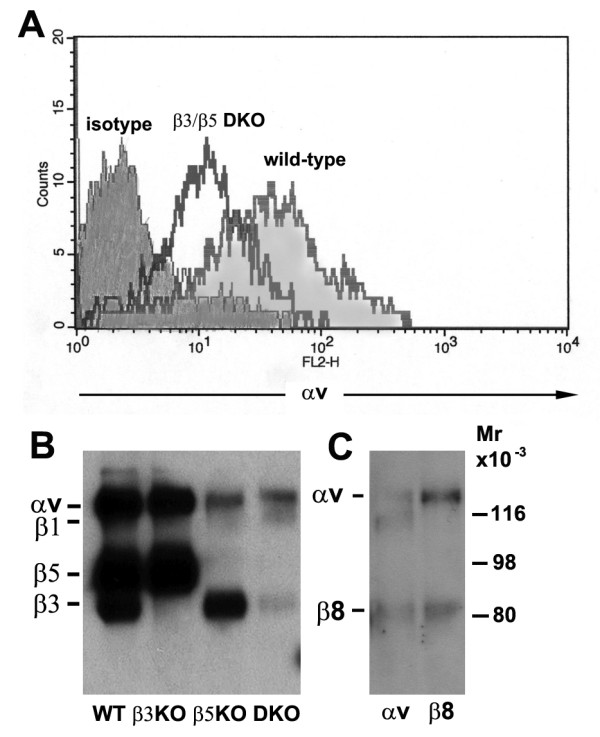
**Characterization of αv integrin expression on microglia derived from wild-type, β3 integrin KO, β5 integrin KO and β3/β5 integrin DKO mice**. A. Flow cytometry analysis on microglia derived from wild-type or β3/β5 integrin DKO mice. Microglia were purified from mixed glial cultures as described in Materials and Methods, and then cultured in serum-free medium on vitronectin. After two days in culture, microglial expression of the αv integrin subunit was analyzed by flow cytometry. Note that β3/β5 integrin DKO mice microglia express the αv integrin subunit, though at much reduced levels compared to wild-type cells. B. Biochemical analysis. An αv integrin imunoprecipitation of wild-type microglia revealed a pattern of three bands: αv (140 kD), β5 (90 kD) and β3 (80 kD). As expected, αv imunoprecipitations of β3 KO microglia showed only two dominant bands: αv and β5, while that on β5 KO microglia showed only two dominant bands: αv and β3. Significantly, αv imunoprecipitations of β3/β5 DKO microglia showed that the αv subunit was still present, though at much reduced levels compared to wild-type cells, and in association with weak levels of two β subunits running at the molecular weights of 110 and 80 kD, which correspond to β1 and β8 integrin subunits, respectively. C. Confirmation that microglia express the αvβ8 integrin. Immunoprecipitations of DKO microglia with a β8 integrin polyclonal antibody detected a pattern of two bands running at 140 kD and 80 KD, that co-migrate with the αv and lower β integrin subunit detected in the αv immunoprecipitation. This confirms that the extra 80 kD band expressed by microglia is the β8 integrin subunit.

Unexpectedly, our experiments revealed that microglia lacking the β3, β5 or both integrins show no defects in their activation responses to vitronectin. As previous pharmacological function-blocking experiments demonstrated that αv integrins are an important mediator of this response [11], this suggests that microglia may express additional αv integrins to mediate this effect. To test this, we performed flow cytometry on β3/β5 DKO microglia to evaluate expression of the αv integrin subunit. This showed that β3/β5 DKO microglia express the αv integrin subunit, albeit at much-reduced levels, approximately 10-15% that of wild-type cells (Figure 4A). To confirm this finding, we next examined this at the biochemical level by performing αv immunoprecipitations on all four strains of microglia: wild-type, β3 KO, β5 KO and β3/β5 DKO. The advantage of this approach is that it reveals all the αv integrin heterodimers expressed by microglial cells, so as well as addressing whether αv integrin is expressed, it also allows us to identify the different β integrin subunits that associate with the αv subunit. Microglia were cultured on vitronectin under serum-free conditions for two days, then cell surface molecules biotinylated and lysates prepared. As shown in Figure 4B, wild-type microglia expressed the αv integrin subunit (140 kD) in association with high levels of the β3 (80 kD) and β5 (90 kD) integrin subunits. As expected, an αv immunoprecipitation of β3 KO microglia revealed only the αv and β5 subunits, while one of β5 KO microglia revealed only the αv and β3 subunits. Significantly, an αv immunoprecipitation of β3/β5 DKO microglia showed that the αv subunit was still present, albeit at very low levels, in association with two different integrin β subunits, with molecular weights of approximately 110 and 80 kD. Based on molecular weight, these can be identified as the β1 and β8 subunits, respectively [21,26,27]. Further immunoprecipitations with a β8 integrin-specific antibody revealed a pattern of two bands, running at approximately 140 and 80 kD, corresponding to the αv and β8 subunits respectively, confirming β8 as the additional β subunit. Levels of microglial β8 expression were not significantly different amongst the different strains of mice (not shown). Thus, these biochemical results support our flow cytometry observations, demonstrating that in addition to αvβ3 and αvβ5, microglia also express low levels of two additional αv integrins, αvβ1 and αvβ8.

### The microglial αvβ8 integrin acts as a functional vitronectin receptor, and αvβ3 is a functional fibronectin receptor

**Figure 5:**
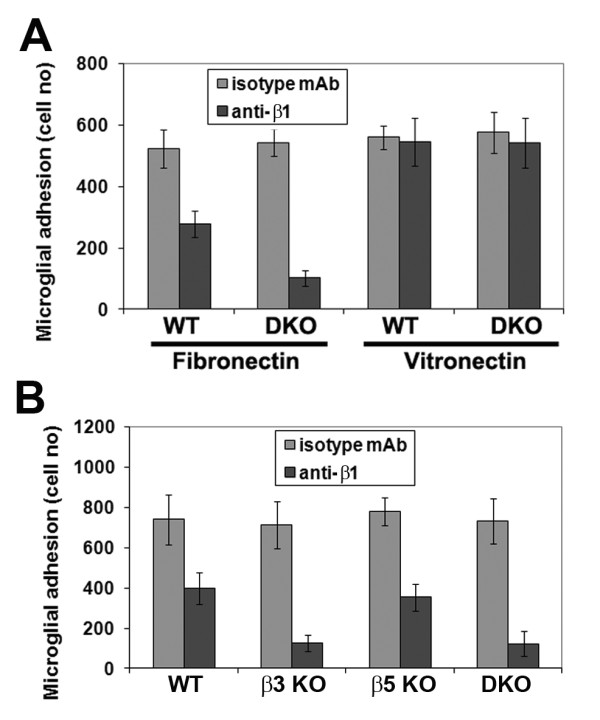
**Examination of the role of β1 integrins in mediating microglial adhesion to vitronectin or fibronectin**. A. Adhesion to vitronectin or fibronectin of microglia derived from wild-type or β3/β5 integrin DKO mice was examined in the presence of a β1 integrin function-blocking antibody. Adhesion is expressed as the number of cells adherent within a given field of view after 30 minutes of cell adhesion. All points represent the mean ± SEM of three experiments. Note that β1 integrin blockade had no impact on the adhesion of wild-type or β3/β5 integrin DKO microglia to vitronectin, but that it inhibited microglial adhesion to fibronectin by approximately 50% (wild-type) or greater than 80% (DKO). B. Adhesion to fibronectin of microglia derived from wild-type, β3 KO, β5 KO or β3/β5 integrin DKO mice was examined in the presence of a β1 integrin function-blocking antibody. Note that β1 integrin blockade of wild-type or β5 integrin KO microglia resulted in approximately 50% inhibition of adhesion to fibronectin, but in β3 integrin null or DKO microglia, the inhibition was greater than 80%.

While αvβ1 integrin is a well described functional vitronectin receptor [28], it is less clear whether the αvβ8 integrin also fulfils this role. To investigate whether αvβ8 is a functional vitronectin receptor in microglia, we examined the effect of function-blocking anti-β1 integrin antibodies on microglial adhesion to vitronectin. Under these conditions, the β3/β5 integrin DKO microglia have only one potential αv integrin available to mediate adhesion to vitronectin, namely αvβ8. As shown in Figure 5, in 30 minute adhesion assays, β1 integrin blockade had no impact on the adhesion of wild-type or β3/β5 integrin DKO microglia to vitronectin. This shows that in the absence of αvβ1, αvβ3 and αvβ5 integrins, microglia can still attach to vitronectin, suggesting that the αvβ8 integrin serves as a functional vitronectin receptor on microglia. Interestingly, β1 integrin blockade revealed markedly different effects on the ability of wild-type and β3/β5 integrin DKO microglia to adhere to fibronectin. While the adhesion of wild-type microglia was inhibited by approximately 50% (from 523 ± 62 cells under control conditions to 278 ± 43 cells with the anti-β1 antibody, p < 0.01), the adhesion of β3/β5 integrin DKO microglia to fibronectin was inhibited by more than 80% (from 543 ± 45 cells under control conditions to 102 ± 25 cells with the anti-β1 antibody, p < 0.005). Direct comparison of the effect of β1 integrin blockade on wild-type or β3/β5 integrin DKO microglia adhesion to fibronectin was found to be statistically significant (p < 0.005). That β1 integrin blockade only partially inhibits WT microglial adhesion to fibronectin suggests that microglia use other receptors to adhere to this substrate. As β1 integrin blockade is much more effective at blocking the adhesion of β3/β5 integrin DKO microglia to fibronectin, relative to wild-type cells, this suggests that the αvβ3 or αvβ5 integrins may also act as fibronectin receptors. To determine the role of the αvβ3 or αvβ5 integrins in microglial adhesion to fibronectin, we next examined the effect of β1 integrin blockade on the four different strains of microglia (Figure 5B). This showed that β1 integrin blockade resulted in approximately 50% inhibition of adhesion to fibronectin of wild-type (from 740 ± 123 cells to 398 ± 79 cells with anti-β1 antibody, p < 0.02), or β5 integrin null microglia (from 778 ± 69 cells to 355 ± 67 cells with anti-β1 antibody, p < 0.01), but greater than 80% inhibition in β3 integrin null (from 713 ± 116 cells to 126 ± 41 cells with anti-β1 antibody, p < 0.005) or DKO microglia (from 733 ± 112 cells to 123 ± 61 cells with anti-β1 antibody, p < 0.005). Taken together, this suggests that the αvβ3 integrin, but not the αvβ5 integrin, makes a significant contribution to microglial adhesion to fibronectin.

## Discussion

Microglia play a critical role in the CNS by performing immune surveillance and regulating inflammatory processes [1,2], therefore defining the factors that control microglial activation state is of fundamental importance. Evidence suggests that ECM proteins play an important role in this process. In particular, vitronectin, present at high levels in plasma [8], and absent in the normal CNS, is deposited in a perivascular manner in MS tissue [13] and in the mouse model of MS, EAE [11,15]. Consistent with this, we have shown that vitronectin directly promotes microglial activation in vitro [10,11]. The next important question is to identify the microglial receptors that mediate this effect. Having shown previously that microglia express the two vitronectin receptors, αvβ3 and αvβ5 integrins [10,23], the purpose of the current study was to investigate the contribution of these two integrins in microglial activation, and thereby test our hypothesis that absence of both αvβ3 and αvβ5 integrins would render microglia unresponsive to vitronectin. Using microglia derived from different strains of mice (wild-type, β3 integrin KO, β5 integrin KO and β3/β5 integrin DKO), different aspects of microglial activation were examined. Surprisingly, when cultured on vitronectin, microglia from the knockout strains showed no obvious defects in adhesion, activation marker or MMP-9 expression, or phagocytosis of vitronectin-coated beads. To investigate the reason for this lack of effect, we examined the expression of other αv integrins. This revealed that microglia also express low levels of the alternative vitronectin receptors, αvβ1 and αvβ8 integrins. This demonstrates that the αvβ3 and αvβ5 integrins are not essential for mediating microglial activation responses to vitronectin, but that microglia use multiple redundant receptors to mediate interactions with this ECM protein.

### Non-essential roles for the αvβ3 and αvβ5 integrins in microglial activation

Evidence gathered from a variety of different cell types supports a role for the αvβ3 and αvβ5 integrins in promoting cellular activation and phagocytic responses. The αvβ3 integrin has been implicated in mediating phagocytosis in monocytes and peripheral macrophages, and the αvβ5 integrin plays a similar role in macrophages and in retinal pigment epithelial (RPE) cells [24,25,29]. Indeed, β5 KO mice develop accelerated age-related blindness as a result of defective phagocytic clearance of old photoreceptor cells in the retina [19]. In addition, αvβ3 plays an essential role in osteoclast function, supported by the fact that osteoclasts in β3 KO mice have defective bone resorption [30]. With this in mind, we were surprised to find that microglia lacking αvβ3 and αvβ5 integrins showed normal adhesion and activation responses to vitronectin, and is further demonstration that the findings of pharmacological blockade studies are not always borne out by the use of genetic KO approaches. This is perhaps best illustrated by the case of the role of αvβ3 and αvβ5 integrins in promoting angiogenesis, in which antibody blockade suggested key roles for these integrins in angiogenesis [31,32], but mice lacking these integrins display no apparent angiogenic defect [16], actually displaying an enhanced angiogenic response in tumor growth [33].

### Redundancy of microglial αv integrins

Our data show that the αvβ3 and αvβ5 integrins comprise the major fraction of total αv integrins expressed by microglia. However, we have found that microglia also express two other vitronectin receptors, the αvβ1 and αvβ8 integrins, though at appreciably lower levels than αvβ3 and αvβ5. What is surprising about our data is that despite lacking the two abundant vitronectin receptors, β3/β5 DKO microglia show no obvious defects in adhesion to vitronectin, or in the subsequent activation responses. This is in stark contrast to our finding with brain endothelial cells, where absence of the αvβ3 integrin leaves the cells totally unable to attach to vitronectin [34]. This clearly demonstrates that the αvβ3 and αvβ5 integrins are not essential for mediating microglial activation responses to vitronectin, highlighting the redundancy of microglial vitronectin receptors. In this light it is informative to compare the αv integrin expression profile of microglia with other CNS cell types. Both neural stem cells and oligodendrocyte precursor cells also express the four αv integrins expressed by microglia, αvβ1, αvβ3, αvβ5 and αvβ8 [21,27,35], and it is interesting to note that all three cell types have the capacity to migrate considerable distances, even in the adult CNS. In contrast, astrocytes, which are far less motile, express only αvβ5 and αvβ8 [18], while brain endothelial cells express just the αvβ3 integrin, and then only when actively undergoing angiogenesis [34,36,37].

### Regulation of αv integrin heterodimer formation

Integrins comprise a family of αβ heterodimers, composed of 11 different α and 9 different β subunits. In light of the potential to form up to 99 potential different αβ heterodimers, in reality, only 24 heterodimers have been identified [26]. We have shown that microglia express four different αvβ heterodimers, with high levels of αvβ3 and αvβ5, and much lower levels of αvβ1 and αvβ8. This begs the question: what regulates the coupling and abundance of each of the different αv heterodimers? It has been previously suggested that the αv integrin subunit is produced in excess, and its cell surface appearance is limited by the transcription level of the associated β subunits [38]. An alternative mechanism would be that the αv subunit is produced in a fixed amount, and that loss of one or more β subunits (e.g.: β3) would automatically lead to compensatory upregulation of the other β subunits (e.g.: β8). Our data would appear to support the first mechanism, because loss of both of the major β subunits β3 and β5 in microglia did not result in any compensatory upregulation of αvβ1 or αvβ8; rather the total amount of the αv integrin subunit was massively reduced.

## Conclusions

The aim of this study was to define the role of the αvβ3 and αvβ5 integrins im mediating microglial activation responses to vitronectin. Microglia from β3, β5, or β3/β5 knockout strains showed no defects in adhesion, activation marker expression, MMP-9 induction, or phagocytosis of vitronectin-coated beads. Flow cytometry and biochemical analysis revealed that microglia also express low levels of the alternative vitronectin receptors, αvβ1 and αvβ8 integrins. Taken together, we conclude that the αvβ3 and αvβ5 integrins are not essential for mediating microglial activation responses to vitronectin, but that microglia employ multiple receptor systems to mediate interactions with vitronectin. On embarking on these studies, we were hopeful that identification of a single vitronectin receptor would lead to potential therapeutic targets for blocking microglial activation. The outcome of the current study suggests that pinpoint targeting of single αv integrins will not be productive, but rather a broad-spectrum blockade aimed at targeting all αv integrins is more likely to be successful.

## Competing interests

The authors declare that they have no competing interests.

## Authors' contributions

JVW genotyped the KO mice strains, prepared the cell cultures, and contributed to drafting the manuscript. AB genotyped the mice, ran the gel zymography and contributed to drafting the manuscript. UT performed the flow cytometry and contributed to drafting the manuscript. RM conceived of the study, performed the biochemical analysis, and drafted the manuscript. All authors read and approved the final manuscript.
